# The Road Not Taken: What Developmental Psychology Might Learn From Darwin’s Insights Concerning Sexual Selection

**DOI:** 10.3389/fpsyg.2022.900799

**Published:** 2022-05-23

**Authors:** Stefan M. M. Goetz, Carol Cronin Weisfeld, Glenn E. Weisfeld

**Affiliations:** ^1^Peace Research Institute Oslo, Oslo, Norway; ^2^Department of Psychology, University of Detroit Mercy, Detroit, MI, United States; ^3^Department of Psychology, Wayne State University, Detroit, MI, United States

**Keywords:** sexual selection, sex differences, developmental psychology, developmental pedagogy, stage theories, adaptationism

## Abstract

Developmental Psychology is the branch of psychology that studies, not only human behavior, but how and why human behavior changes over time. This essay seeks to review to what extent Developmental Psychology has failed to perceive human behavior through the lens of evolutionary theory in general, and in particular sexual selection as first described by Darwin and later elaborated on by many, including Robert Trivers and Geoffrey Miller; the essay asserts that this failure has resulted in many wrong turns and missed opportunities. In some cases, major developmental theorists (e.g., Freud, Erikson) were bedeviled by sex-based differences which they saw but could not explain and which compromised the parsimony of their stage theories. In the case of stage theories of moral development, some major theorists (e.g., Piaget, Kohlberg) were able to offer simpler explanations of moral development only by limiting their studies to male subjects. And, while Developmental Psychology textbooks thoroughly describe sex differences in the timing of morphological changes in puberty, writers seldom discuss why the timing is different in the two sexes, universally, and functionally. On the other hand, several domains of developmental focus, including play, mate choice, parenting, and spatial cognition, have seen successful research efforts that utilized sexually selected predispositions as foundational assumptions. The essay concludes with a discussion of how a more evolutionary and functional view of human behavior might move the field of Developmental Psychology to an even more robust and accurate understanding of how humans change over the course of a lifetime.

## Introduction

As we mark 150 years since Darwin’s publication of *The Descent of Man, and Selection in Relation to Sex*, it is fitting that we reflect on Psychology’s limited ability to benefit from Darwin’s insights. Eloquent essays have been written on the broad failure to integrate Evolutionary Theory into the Social Sciences, including Psychology (Tinbergen, 1963; Pinker, 2004; Barkow, 2006; Segerstråle, 2006). Expectations for integrating Evolutionary Theory might have been particularly high for Developmental Psychology, which aims to explain changes in human behavior over the lifespan; those expectations have not been met either (MacDonald, 1988; Charlesworth, 1992). Looking at Sexual Selection as contrasted with Evolutionary Theory more generally, one is even more struck by Developmental Psychology’s stubborn resistance; Sexual Selection has been a fearsome lightning rod indeed.

There are many reasons for Developmental Psychology’s failure to integrate ideas from Sexual Selection Theory. These causes include Western philosophy’s history of dualism, a combination, culturally, of ignorance and arrogance about sexual matters, a focus on practical measures to improve people’s lives (meliorism), and suspicion of biology coming from both United States political wings. The result has been an emphasis on socialization and other culture-specific environmental factors in the development of human behavior, as opposed to species-wide biological factors. Above all, developmental psychology has failed to explore functional explanations for universal human behaviors and sex differences. Though the reasons for this failure are numerous, one prominent cause may be the misperception that because functional explanations imply genetic causation, extragenetic influences on phenotype development are relatively trivial, a position which is inconsistent with a dynamic systems theory perspective (Lickliter and Honeycutt, 2003; Badcock, 2012; Al-Shawaf et al., 2021; Narvaez et al., 2021)^1^. While a focus on ultimate explanations can lead to an overemphasis on biological causation (Narvaez et al., 2021), it is important to note also the benefits of theory in building a coherent science (Eronen and Bringmann, 2021). Like many before us (e.g., Ploeger et al., 2008; Bjorklund, 2018), we believe that a Darwinian approach holds great potential, in conjunction with other theoretical approaches (e.g., developmental systems theory), as a metatheory for a mature developmental psychological science.

This essay will not dwell on why there is this powerful bias toward nurture-based explanation; the dynamics have been amply described in prior work. Rather, this essay will briefly summarize some basic ideas from Sexual Selection Theory with relevance for Developmental Psychology. The essay will then describe the path taken by Developmental Psychology, as reflected in major textbooks used in higher education courses, and point out failures in theory and research resulting, arguably, from failing to recognize the importance of Sexual Selection. The essay will end with examples of successful integration of Sexual Selection insights into developmental research and call for more such efforts.

### Sexual Selection Theory: Concepts Relevant to Developmental Psychology

Darwin was puzzled by the consistency of the sex difference in animals whereby males compete for sexual access to females. In 1972, Trivers solved the conundrum by providing a theory to explain this pattern (Trivers, 1972). He recognized that the female is usually the more nurturant sex, and the males compete for access to this essential, limiting asset, often resulting in the evolution of larger, aggressive males. Males can reproduce merely by inseminating a female, whereas in mammals most notably, the female must invest much more energy, providing internal fertilization, pregnancy, parturition, and lactation. Therefore, females are selected to be judicious in choosing a mate, a pattern discernable in humans as well as most other mammals. Mammals’ internal fertilization also means that pair-bonding males are prone to uncertainty about paternity, so selection favors males’ guarding their mates against sexual interlopers (Mealey, 2000). Correspondingly, pair-bonding females’ interests are more threatened by desertion by their mate.

Evolutionary theorists seek functional explanations for species-wide traits, including sex differences in behavior. They are interested in these “why” (ultimate; Mayr, 1961; MacDougall-Shackleton, 2011) questions that even intrigue young children but don’t seem to puzzle mainstream psychologists. Developmental psychology textbooks seem not to recognize the extent to which human behavioral sex differences can be explained by extensions of what Trivers calls the theory of sexual selection and parental investment.

While some have questioned the usefulness of posing “why” questions (e.g., Laland et al., 2011), we see value in their inclusion. First, functional explanations provide heuristic and inductive value for hypothesis generation at the proximate (how) level of explanation. Knowing that the heart acts as a pump immediately suggests how components of the system might interrelate. Second, functional explanations can help define categories of behavior providing ontological clarity. For instance, the form of a behavior often belies its function. Both an infant’s smile and cry, while differing in form (and state) function to reduce the distance between him and his caregiver. Likewise, the form of status-seeking behavior can include both prosocial and antisocial behaviors, yet serve functionally identical purposes (Hawley, 1999, 2016). Functional explanations can also distinguish between behaviors that share form (e.g., maternal aggression vs. reactive aggression). Third, functional explanations can provide an additional type of support (or lack thereof). Both proximate and ultimate explanation ought to be concordant. Forth, a functional understanding can improve targeting of developmental guidance (Ellis et al., 2012). Finally, our understanding of a given phenomenon is simply much richer if both levels of analysis are addressed. Knowing that male birds sing due to rising testosterone levels during the breeding season (proximate explanation) is categorically different than knowing that male birds sing to attract mates (ultimate explanation), but knowing both can be quite satisfying. See MacDougall-Shackleton (2011) for further discussion.

Returning to ultimate explanations derived from Sexual Selection theory, patterns of size dimorphism can be understood from this perspective. For instance, as in other species with male sexual competition, men are taller and stronger than women on average in all populations, maturing a year or two after girls. Adolescent boys and men are more aggressive and competitive than girls and women across cultures (e.g., Schlegel, 1995). Around the world, boys exhibit more play fighting than girls in order to practice combat and to establish dominance relations.

Men’s greater desire for status and resources for acquiring mates is linked to men’s higher violent crime rates (Wilson and Daly, 1985) and putatively responsible for men’s greater strength, especially upper body strength useful for fighting. Whereas men often react to a threat of attack by mobilizing physiologically for a fight, women and other female mammals often act to protect their young or to seek help, a reaction partly mediated by oxytocin (Taylor, 2006).

The sex differentiation in reproductive behavior is mediated in part by hormones. Accordingly, Sexual Selection Theory has come to incorporate elements of endocrinology, utilizing more elements of endocrinology than were available to Darwin.

Female mammals are exposed to little or no androgenic influence before birth, resulting in less play fighting than males. Girls with abnormally high exposure to androgens before birth show more play fighting than control girls, and less interest in doll play and marriage (Hooven, 2021). At puberty, interest in babies rises in both sexes, especially among females (Goldberg et al., 1982). At puberty, girls are feminized, mostly by estrogens. In both sexes, high levels of sex-typical gonadal hormones contribute to sex differentiation and hence in traditional evolutionary models are assumed to contribute to sexual attractiveness (Law Smith et al., 2006; Lidborg et al., 2022).

A woman’s level of several parental hormones during pregnancy or after birth is correlated with her bonding and nurturant tendencies. In mammals, maternal motivation is driven by hormones, exposure to young, filial emotional displays, competence, learning, and imitation. Men’s level of testosterone falls, and their level of prolactin rises when their mate becomes pregnant and when the baby is born (Storey et al., 2000). The greater these changes, the more paternal behavior the father exhibits (e.g., Gettler et al., 2011). Thus, parental behavior is shaped partly by hormones and experience in both sexes, but the hormones differ.

The parental roles are somewhat specialized by gender. Around the world, fathers tend to defend the nuclear family from attack (Opie et al., 2013), and fathers are expected to be more productive economically. Women, however, perform labor outside the home, prehistorically having gathered plant food (Friedl, 1975). Tribal cultures exhibit clear sex differentiation of labor, avoiding disputes over task assignment and redundancy and gaps in training during childhood.

Girls living in stressful or father-absent homes tend to reach menarche earlier than controls (Ellis, 2004). Boys in abusive homes and who possess a certain gene on the X chromosome, are at risk of antisocial behavior, and more at risk than girls. Abuse, including sexual abuse and homicide, is more likely when an unrelated male, such as a boyfriend or stepfather, resides in the home (Daly and Wilson, 1988). In general, development by any measure is better when both biological parents are in the home.

Many other human reproductive behaviors do not differ between the sexes but are universal and hence may have an evolved basis. Almost all tribal cultures are extended-family cultures in which three generations live together or in proximity. These universals are of interest because of their relevance to survival and reproductive success. Comparatively, biparental species tend to show marked reductions in sexual dimorphism compared to promiscuous or polygynous ones, a trend visible in our hominin ancestors (Grabowski et al., 2015). Pair-bonding emotions e.g., amorousness seems to be monotropic and qualitatively isomorphic and correlates of marital satisfaction are shared (Weisfeld et al., 2018). Both sexes compete for dominance, albeit males more so (Weisfeld, 1986), with forms of aggression also differing (Campbell, 1999; Pellegrini and Archer, 2005). Correlates of divorce, such as infertility are shared between the sexes, although earnings, financial independence, and infidelity differentially impact divorce across the sexes (Betzig, 1989). By many indications, including the fact that almost all people, around the world, seek to marry, marriage is a reproductive adaptation (Weisfeld et al., 2018).

### Teaching Life-Span Developmental Psychology

We three authors have altogether taught various courses in Developmental Psychology for over 85 years. In teaching Developmental Psychology, we have sought to use texts that integrate causal explanations for behavior, reasoning that heredity and socialization interact throughout the lifespan. That is, after all, the charge of Developmental Psychology, understood from the time that the journal by that name began publication in the 1960’s: *to explain changes in behavior as a function of time.* As the first editor, Boyd R. McCandless, wrote, this would surely require integrating nature and nurture, including accounting for sex differences (McCandless, 1969). Yet published research and textbook writing certainly veered off that path early and often. Our own difficulty in finding truly integrative Developmental Psychology textbooks is reflected in the data provided in Table 1. In preparing this table, we created a list of concepts from Sexual Selection Theory that are relevant to Developmental Psychology, and we searched the subject index from 10 mainstream texts to see if the topic appeared (Fitzgerald, 1986; Berk, 2001; Rice, 2001; Broderick and Blewitt, 2003; Boyd and Bee, 2009; Feldman, 2009; Sigelman and Rider, 2018; Kuther, 2020; Kail and Cavanaugh, 2021; Kraynock et al., 2022). In many cases, whether or not the topic was found in the index, we searched further in the text to see whether the connection to sexual selection was made; if yes, we marked it as included, and if not, we marked it not included. For example, “breastfeeding” was mentioned in some books in terms of how long it’s recommended by U.S. pediatricians, but the functional reasoning, in terms of how milk varies with the nursling’s age and gender, was not presented. We included one text published before the year 2000 (Fitzgerald, 1986, which never went into second edition), because we considered it to include good integrative reasoning for its time. The text box below enumerates, for each concept, what percentage of the 10 texts made connections to the relevant concepts from Sexual Selection Theory.

**TABLE 1:** Topics for sexual selection usually missing from mainstream texts on developmental psychology.

Devlopmental topic	Missing relevant sexual selection concepts (Percentage of 10 texts* including concept)
Basic theories and research methods	(90%) Importance of cross-cultural research (30%) Research on foragers, or hominid evolution (90%) Naturalistic observation
Genes, environment, and development	(0%) Sexual and asexual reproduction (20%) Evolution of sex chromosomes; genomic imprinting (0%) Sexual selection pressures on the sexes at different ages
Prenatal development and birth	(0%) Evolutionary theories of post-partum depression (0%) Primate pattern of nocturnal births (0%) Evolution of lactation, weaning conflict, variation of milk with developmental age and sex (20%) Role of pregnancy and lactation in lowering risk of breast cancer for women
Physical growth (brain, body, and health)	(10%) Function of sex differences in maturation rate (0%) Function of secondary sex differences (hairiness, etc.) (0%) Pubertal behavioral changes and their relevance to sexual selection: Competitiveness, nurturance, libido, risk-taking, pair-bonding, jealousy (0%) Role of obesity and family stress in accelerating menarche
Sensation and perception	(0%) Function of general female superiority in sensation (10%) Evolution of sex differences in visual-spatial skills
Cognition and intelligence	(0%) Role of parents and older peers as teachers of sex-specific skills (50%) Role of elders across cultures as repositories of knowledge
Language and education	(10%) Implications and consequences of cross-national decline in male education and achievement
Self and personality	(10%) Sex differences in achievement motivation (0%) Role of testosterone in raising/lowering self-confidence
Sex and gender roles	(40%) Integration of role of hormones throughout lifespan (e.g., heterochrony) (70%) Life expectancy differences (e.g., males die earlier, due to both hormonal and lifestyle factors) (0%) Grandmother hypothesis (females may live longer to increase reproductive success of their own offspring) (50%) Occupational sex differences (70%) Genetic, olfactory, and hormonal correlates related to sexual orientation
Moral development	(70%) Major theories and their difficulties with explaining gender differences and cultural differences
Attachment, social relationships, and family	(10%) Role of oxytocin in attachment (0%) Sex similarities and differences in hormones and brain areas involved in parenting behaviors (100%) Gender-related similarities and differences in play (10%) Sex differences in mate criteria (10%) Factors affecting marriage rate and age at first marriage (0%) Advantages of biological father being present
Psychopathology	(10%) Sex differences in internalizing vs. externalizing pathologies (10%) Sex differences in perpetrators and victims of homicide, rape
Aging, death, and grieving	(70%) Sex differences in reactions to death of a spouse

As is apparent, 9 of the 10 texts acknowledge that cross-cultural research and naturalistic observation are important methodologies in Developmental Psychology. Only 3 books discussed human evolution as a source of useful information. All 10 of the books describe gender-related similarities and differences in play, but explaining why they occur cross-culturally varied widely, in terms of integrating biological and socialization explanations. For example, Boyd and Bee (2009) present sex differences in toddlers’ play as the product of males’ identity development; in contrast, Fitzgerald (1986) and Kuther (2020) discuss the roles of prenatal hormones and environmental effects as inseparable influences. Out of the 36 concepts, 14 are not mentioned at all, and 9 are mentioned in one text. In addition to what appears in Table 1, the term Sexual Selection Theory (Darwin, 1871) was not found in any text; Parental Investment Theory (Trivers, 1972) was presented incorrectly in 1 text. As Surbey put it in her 1998 essay, our intention is not to admonish teachers of Developmental Psychology, but, rather, to encourage teachers to be open to integrating additional information into the way they think about development and the way they present it to students.

### What if? How Theories Might Have Benefited From Sexual Selection Ideas

Stage theories are a major contribution from Developmental Psychology, as they function as path models, aiding our ability to predict behavioral changes over time. Dozens of stage theories have been proposed, but only a handful are generally familiar: those proposed by Freud (psychosexual development); Erikson (psychosocial development); Piaget (cognitive development); and Kohlberg (moral development). In each case, save perhaps Piaget’s, the theorist was handicapped by a failure to deal with sex differences.

Freud’s great contribution may be seeing that much human behavior is driven by unconscious forces (Miller, 2016); but Freud’s developmental stages (oral, anal, phallic, latency, genital) are generally not supported by empirical data (Rutter, 1971). Indeed, Freud admitted his frustration at not being able to fit female experience into his theory of the Oedipal Complex—through which a boy’s castration anxiety leads to identification with the father figure and elaborated moral functioning via a mature superego. Eventually, Freud (1925) proposed (1925) that because females never experience castration anxiety, females have compromised superegos, and therefore females’ moral reasoning and civil functioning can never be as mature as what males offer. As Gilligan concluded, “Thus a problem in theory became cast as a problem in women’s development, and the problem in women’s development was located in their experience of relationships” (Gilligan, 1982, p. 7). Several neoanalytic theorists (e.g., Chodorow, 1974) endeavored to reframe Freud’s theory in a way that does less violence to female development, by focusing less on anatomical differences and more on sex role differences.

One major example of such reframing is seen in the stage theory of Erikson (1980), who departed from Freud’s focus on anatomy and sex, again turning to relationships. But Erikson’s fifth psychosocial stage (identity vs. role confusion) and sixth stage (intimacy vs. isolation) seemed to lose their clarity when he discussed females; as Gilligan (1982) pointed out, identity and intimacy may be simultaneous developmental processes, and this seems especially so for females. Gilligan’s insights into weaknesses in Eriksonian theory make even more functional sense if one considers that females everywhere tend to marry at a younger age than males do partially due to limitations on female reproductive potential.

Piaget (1936, 1945) constructed a theory of cognitive development in children and adolescence that has largely stood the test of time, cross-culturally (Bjorklund, 1995; Miller, 2016) and provided the groundwork for research on adult cognition (Sinnott, 1996). As MacDonald observes, Flavell once commented that, “if one wants to understand how Piaget conceived the child, one simply has to ask how evolution would have designed an optimal learning machine” (MacDonald, 1988, p. 14). MacDonald points to the young child’s intrinsic motivation and responsiveness to general features of the environment. It would be difficult to argue that Piaget’s cognitive development theory would have substantially benefited from his paying more attention to Sexual Selection. It is a different matter, however, with Piaget’s theory of moral development (Piaget, 1932). Both Piaget and his intellectual heir, Kohlberg, were so bedeviled by gender differences in their findings that they chose to do most, if not all, of their morality research on males (Gilligan, 1982). Baron-Cohen (2005) addressed gender differences in social decision-making directly, when he modified his own theory of mindreading to make a “model of empathizing.” This model describes development in children up to age four, but its predictions fit with patterns of findings across ages and cultures. Both sexes are more likely to empathize than to systemize. However, males, on average, systemize more (attend to physical detail, construct, and organize systems) than females do. Females empathize (use eye contact, decode non-verbal expression, and respond empathetically) more than males do. Baron-Cohen (2005, p. 481) concluded that, “From an evolutionary perspective, sex difference in empathizing and systemizing are likely to have been shaped by sexual selection and follow, at least in part, from sex differences in reproductive strategies.” More research is warranted, but this is a promising start toward addressing questions about moral development.

One could argue that early theorists were anxious to see Developmental Psychology recognized as an independent science, distinct from the biological disciplines that were refining their methods at the same time (Charlesworth, 1992). But, once the path was defined as one that did not include biological influences, most developmentalists marched down the path without looking back, leaving the landscape littered with missed opportunities. Surely important opportunities were missed when stage theorists failed to recognize the implications of sexually dimorphic characteristics (often related to Life History processes such as gestation, lactation, and weaning) seen throughout human development.

### What if? How Research Might Have Benefited From Sexual Selection Ideas

Generally, discussion of human sex differences has been met with resistance from Developmental Psychologists. Consider that the number of citations (according to Google Scholar) for Maccoby and Jacklin (1974) volume *The Psychology of Sex Differences* is close to 15,000. The book emphasizes socialization factors involved in sex differences in most behaviors, including aggression. By 1998, Maccoby (1998) had reconsidered biology’s role, describing an interactive process whereby biological factors nudged children toward sex segregation, which increased sex differences in aggression, etc. Yet despite the earlier edition being twice as old, it has garnered five times the number of citations. Similarly, books by Mealey (2000); Lippa (2005), and Geary (2020) have not found wide acceptance within Developmental Psychology.

Typically, Developmental Psychology textbooks document sex differences in the timing of morphological changes in puberty. With few exceptions (Weisfeld and Billings, 1988; Weisfeld, 1999), writers seldom discuss why the timing is different in the two sexes, universally, and functionally. Boys retain their child-like morphology longer, to gain experience and strength before their physical characteristics engender competitive responses from mature males (see Dunsworth, 2020, for an alternative, proximate explanation). Darwin had seen this sex difference in functional terms, when he described how young boys around the world practice wrestling, often not marrying before the age of twenty “as before that age they cannot conquer their rivals” (Darwin, 1871, p. 872). Only a handful of developmentalists (Aldis, 1976; LaFrenière, 2013) have pointed out that, while boys do more rough-and-tumble play, both sexes engage equally in chase games, honing escape abilities—demonstrating the utility of extending Sexual Selection Theory to understand functional patterns of universal and divergent patterns of play.

Again, the play literature tends to see bullying behavior in cases of what is arguably normal playfighting in preadolescent children. Weisfeld and Weisfeld (2013) refer to the work of Aldis (1976) who described playfighting between siblings as the typical scenario, where actors represent different ages and sizes, and where the larger actor frequently switches roles to let the smaller actor “win”—all with smiles and laughter as expressions of functional play. Since the work of Olweus (1978) this has been confused with bullying. The result is not only poorly conceptualized research leading to wasted resources and ineffectual programs (Merrell et al., 2008); the confusion has also led to reducing recess and punishing behaviors that may be developmentally normal and functional.

### Where Has Sexual Selection Theory Benefited Developmental Psychology?

One can find intriguing examples of research that recognizes sex differences and explores those differences as part of the Developmental Psychologist’s challenge. Both Charlesworth and his student, LaFrenière, did observational research on children’s play and observed marked sex differences (Charlesworth and Lafreniere, 1983). Applying a functional lens, LaFrenière et al. (2002) were able to document those sex differences in 10 cultures showing that girls expressed more empathy and social competence, while boys’ behavior exhibited more physical aggression and dominance. Later LaFrenière (2013, p. 183) would write, “For boys especially, rough-and-tumble play in early childhood provides a scaffold for learning emotion regulation skills related to managing anger and aggression.” Thus, LaFrenière identified both an ultimate function for the behavior (male-male competition for future reproductive success) and a proximate function (social success with peers in childhood). As Mealey had observed earlier, “…the existence of two physical sexes virtually ensures the existence of two psychological sexes” (Mealey, 2000, p. 40).

Recognizing sex differences is fundamental for understanding the development of visual-spatial skills (Maccoby and Jacklin, 1974; Hoyenga and Hoyenga, 1993; Kimura, 1999; Geary, 2020). Silverman and Eals (1992) and Eals and Silverman (1994), in a series of observational experiments, found that male undergraduate students were superior at wayfinding, while female undergrads were superior at remembering objects and their placement in a two-dimensional array. They explained this through the “hunter-gatherer hypothesis” which attributed the differences to survival-related skills developed in humans’ hunter-gatherer past and inherited as predispositions seen in males and females today. As hunters, the theory explains, men utilized three-dimensional skills traveling great distances in search of prey and then finding their way back home. As gatherers, women covered shorter distances but remembered where they saw signs of edible plants on the ground—so that they could return and harvest what was ready to eat. Thus, the differences in visual-spatial skills are highly specialized, for functional reasons. Later, this theory was confirmed with data from 40 countries (Silverman et al., 2007). Interested in when these differences appear developmentally, Choi and Silverman (2003) designed experiments showing that some sex differences are apparent in 9-year-olds, but elaborated sex differences do not appear until about 3 years later. This suggests roles both for pubertal hormones, and for refining differentiated strategies through practice.

Life History Theory, developed in evolutionary ecology in the 1950’s (Stearns, 1976), and later applied to the human lifecycle (Hill and Kaplan, 1999), attempts to explain life-history strategies (e.g., sexual debut) in terms of selective forces, some of which challenge the sexes differentially. Falling within Life History Theory, the concept of sex ratio, the ratio of males to females, affects a wide range of behaviors. While the human primary (before birth) and secondary (at birth) sex ratios are approximately 1:1, the tertiary sex ratio, by early adulthood, is skewed toward females, because males often suffer higher mortality in childhood and adolescence, partially due to intrasexual competition (Mealey, 2000) and “risky behavior” (Daly and Wilson, 1983). Being the scarcer sex, males are then better able to impose their mating priorities. When females are in short supply, the converse applies. For instance, Pollet and Nettle (2008) did archival research on a U.S. population with low numbers of women; these women were able to marry young and marry well, securing husbands who had a higher SES than competing males. Conversely, when young males were scarce, they were less likely to be married, presumably because they were better positioned to pursue short-term mating; however, a higher proportion of these scarce men are married later in life (Kruger and Schlemmer, 2009). This topic deserves more research, especially given current patterns of sex differences in educational attainment (US Census Bureau, 2020), considered alongside tendencies for homophily and hypergamy (c.f., Sassler and Lichter, 2020). The sex ratio is skewed even more when fewer males present as attractive choices. Furthermore, patterns of marriage mobility—female educational hypergamy and male hypogamy (marrying someone with more vs. less education)—are no longer apparent (Qian, 2017; Van Bavel et al., 2018). Data from 28 countries drawn from the European Social Survey indicate that women, rather than remaining unpartnered, will often chose to “cast a wider net,” partnering with men whose socioeconomic status falls short of their own (De Hauw et al., 2017). Similar patterns have emerged in Latin America (Esteve et al., 2016) and the United States (Qian, 2017; Lichter and Qian, 2019).

## Conclusion

Many books document human sex differences ably (e.g., Maccoby and Jacklin, 1974; Hoyenga and Hoyenga, 1993; Mealey, 2000; Lippa, 2005; Geary, 2020). We see heuristic value when a book provides, not just a massive listing of such sex differences, but a story told by the differences. It is the “orderliness” of behavior that is of interest, as Archer (1992) put it. We benefit most from research that recognizes patterns in sex differences and offers an overarching explanatory theory.

We need honest discussion regarding sex roles and implications for salaries and work environments, while simultaneously avoiding rigid normative judgments and outright dismissal of potential bias. Sex segregation in the labor force and gender inequality in pay continues, with the gender gap narrowing slowly in most countries that provide relevant data (Ponthieux and Meurs, 2015). One could argue for breaking down all professional sex segregation; but a stronger argument might be made for raising salaries of predominantly female occupations and reducing barriers in the workplace, so that atypical boys and girls feel comfortable pursuing divergent paths.

For those Developmental Psychologists who would like to read more about Evolutionary Theory and its application to human development, particularly in childhood, *The Origins of Human Nature* (Bjorklund and Pellegrini, 2002) is an important introduction, as are *Human Infancy* (Freedman, 1974), *Ethology and Human Development* (Archer, 1992), and *Adaptive Origins* (LaFrenière, 2010; see also Geary and Bjorklund, 2000; Salmon and Shackelford, 2007; Ellis et al., 2009; Konner, 2010; Geary and Berch, 2016; Bjorklund, 2020; Geary, 2020; Hart and Bjorklund, 2022). Many articles offer good introductions to Sexual Selection theory and its relevance to human development (e.g., Buss, 1995; Miller, 1998; Bjorklund and Pellegrini, 2000; Schmitt, 2005; Puts, 2016; Wilson et al., 2017).

What might be gained by incorporating Darwin’s insights into our work in Developmental Psychology? More complete understanding? More satisfying understanding? Better positioning for making decisions on a personal and societal level? More of a sound basis for arguing for equality of opportunity if we are so inclined? A sounder footing for educating the next generations of teachers, nurses, psychologists, and others who study Developmental Psychology? We argue, simply, yes.

## Author Contributions

SG, GW, and CW conceived of the manuscript idea. GW designed the manuscript outline. GW and CW prepared the first draft. SG restructured and added additional content in the subsequent drafts, proofread, formatted, and submitted the manuscript. All authors contributed to the article and approved the submitted version.

## Conflict of Interest

The authors declare that the research was conducted in the absence of any commercial or financial relationships that could be construed as a potential conflict of interest.

## Publisher’s Note

All claims expressed in this article are solely those of the authors and do not necessarily represent those of their affiliated organizations, or those of the publisher, the editors and the reviewers. Any product that may be evaluated in this article, or claim that may be made by its manufacturer, is not guaranteed or endorsed by the publisher.

## Footnotes

1 It is important to note that a functional explanation does not imply (hard) genetic determinism. While it does posit selection of genes, and a role of genes in development, it does not speak to the questions of canalization, that is robustness or plasticity, of phenotypes. It merely asserts some role of genetic causation and can explain species-typical patterns of behavior. It has long been observed that extragenetic developmental adjustments can alter behavioral phenotypes. For instance, Kuo (1960) demonstrated that breed-typical behavior could be extinguished via developmental adjustments.
